# Experimental model of ventilator-induced lung injury induces proteomic signatures of neurodegeneration in the hippocampus

**DOI:** 10.1186/s40635-026-00981-7

**Published:** 2026-09-29

**Authors:** Kevin D. Winzey, Samuel Guzman, Edward Yang, Debbie Moreira, Xingyu Wang, Xuesong Gu, Simon T. Dillon, E. Wesley Ely, S. Ananth Karumanchi, Towia A. Libermann, Shouri Lahiri

**Affiliations:** 1 Department of Neurology, Cedars-Sinai Medical Center, Los Angeles, CA USA; 2 Division of Interdisciplinary Medicine and Biotechnology, Department of Medicine, Beth Israel Deaconess Medical Center, Boston, MA USA; 3 Proteomics, Bioinformatics and Systems Biology Center, Beth Israel Deaconess Medical Center Genomics, Boston, MA USA; 4 Brain Dysfunction, Survivorship (CIBS) Center, Department of Pulmonary and Critical Care Medicine, Veteran’s Affairs Tennessee Valley Geriatric Research Education and Clinical Center (GRECC), Critical Illness, Vanderbilt University School of Medicine, Nashville, TN USA; 5 Department of Medicine, Cedars-Sinai Medical Center, Los Angeles, CA USA; 6 Harvard Medical School, Boston, MA USA; 7 Department of Neurosurgery, Cedars-Sinai Medical Center, Los Angeles, CA USA; 8 Department of Biomedical Sciences, Cedars-Sinai Medical Center, Los Angeles, CA USA

**Keywords:** Ventilator-induced lung injury, Neuroinflammation, Hippocampus, Aptamer-based proteomics, Neurodegeneration, Lung-brain axis, Delirium, Critical illness, IL-6, Blood-brain barrier

## Abstract

**Background:**

Mechanical ventilation is associated with acute and long-term cognitive dysfunction, yet the molecular pathways linking ventilator-induced lung injury (VILI) to brain injury remain poorly characterized. We previously demonstrated that peripheral IL-6 signaling mediates delirium-like phenotypes in a murine VILI model. Here, we use unbiased aptamer-based proteomics to determine whether this model exhibits proteomic signatures consistent with neurodegenerative processes in the brain and plasma.

**Methods:**

C57BL/6 mice were subjected to VILI via high tidal volume mechanical ventilation or served as spontaneously breathing (SB) controls. Brain tissue (hippocampal and cortical regions) and plasma were analyzed using the SomaScan proteomic platform (10,778 and 7,307 proteins, respectively). Differential expression, hierarchical clustering, principal component analysis, and Ingenuity Pathway Analysis (IPA) were performed to identify dysregulated proteins, functional networks, and predicted upstream regulators.

**Results:**

SomaScan proteomics identified 253 and 995 significantly altered proteins (*p* < 0.05) between VILI and SB mice in cortical and hippocampal regions, respectively, and 290 in plasma. Hippocampal pathway analysis revealed enrichment of neurodegeneration, cognitive impairment, and neural differentiation/maturation categories, with dysregulation of proteins including CSNK2A1, SNCA, BDNF, PLD3, and TMEM240. Predicted upstream regulators included CTNNB1, NR3C1, SNCA, and PPARGC1A. Plasma proteomics identified coordinated shifts in metabolic, inflammatory, and vascular injury pathways consistent with an IL-6-associated acute phase response, with predicted activation of HNF4A and PPARG. Only 9 proteins, including PLD3, HAVCR2 (TIM-3), MAG, and STMN4, were dysregulated across all three compartments, with brain-restricted proteins MAG and STMN4 detectable in plasma. The hippocampal region demonstrated the most pronounced alterations, underscoring region-specific vulnerability.

**Conclusions:**

VILI induced a dominant hippocampal injury signature enriched for neurodegeneration, cognitive impairment, and disrupted neuronal maturation. Parallel plasma shifts were consistent with inflammatory–metabolic stress and identified a concurrent peripheral response potentially relevant to the lung–brain axis, while detection of brain-restricted proteins in plasma raises the possibility of blood–brain barrier compromise or CNS protein release. Together, these findings support a model in which peripheral lung injury activates coordinated peripheral and hippocampal neuroinflammatory pathways implicated in neurodegenerative biology.

**Supplementary Information:**

The online version contains supplementary material available at https://doi.org/10.1186/s40635-026-00981-7.

## Background

Critical illness is increasingly implicated as a precipitating factor in neurodegenerative conditions [1]. Mechanical ventilation, a hallmark of critical illness, is associated with both acute- and long-term cognitive dysfunction [2, 3]. Delirium, or acute encephalopathy, occurs in 50–75% of patients on mechanical ventilation and is even more prevalent in those with severe lung injury phenotypes, such as acute respiratory distress syndrome [4, 5]. Emerging epidemiological evidence suggests a possible causal link between delirium and incident dementia [6], highlighting delirium prevention and management as critical opportunities to mitigate neurodegenerative processes.

Recently developed murine models recapitulate delirium-like phenotypes in the context of mechanical ventilation [7–9], yet their relevance to long-term neurodegenerative processes remains unclear. In prior studies, we demonstrated a direct pathological role for peripheral IL-6 signaling in mediating neurostructural and behavioral delirium-like phenotypes [7, 8, 10]. Complementary retrospective clinical data validate these findings, showing amelioration of delirium outcomes in critically ill patients treated with a peripheral IL-6 pathway antagonist [11].

In this study, we employ an aptamer-based proteomics platform to perform an unbiased analysis of brain and plasma samples from mice subjected to mechanical ventilator-induced lung injury (VILI) to determine the molecular relevance of this model to long-term neurodegenerative processes. We hypothesize that murine models that recapitulate clinical delirium will similarly demonstrate distinct proteomic signatures indicative of neurodegenerative processes.

## Methods

### Animals

Plasma proteomics was performed on a cohort of 8 female C57BL/6 mice aged 5–8 months. Separate brain proteomics cohorts, composed of 12 (6 male, 6 female) C57BL/6 mice aged 5–8 months (Jackson Laboratory), were used in this study. All animals were kept in ventilated cages at approximately 21 °C, 40–70% humidity, a 12-h light/dark cycle, with food and water available to the animals ad libitum in Cedars-Sinai’s AAALAC-accredited animal facility. Entirely new cohorts of animals were used for this study, and all procedures were approved by Cedars-Sinai’s IACUC (protocol # IACUC007914) prior to any work.

### VILI

VILI was induced as described in our prior work [7–9]. These methods were previously validated as a model of VILI in healthy lungs without concomitant acute lung pathology. Mice received intraperitoneal injection of ketamine (75 mg/kg) and dexmedetomidine (0.5 mg/kg) for anesthesia induction, followed by orotracheal intubation. Subcutaneous saline (0.5 mL) was given immediately prior to intubation to support hydration, and ophthalmic ointment was applied to protect the eyes. VILI was induced by volume-controlled mechanical ventilation using VentElite Small Animal Ventilators (Harvard Apparatus, Holliston, MA, USA) with a high tidal volume (35 cc/kg) at 70 breaths/min and zero positive end-expiratory pressure (PEEP), using ambient room air for 2 h in the supine position. Core temperature was maintained with a 38 °C heating pad (Hallowell EMC, Pittsfield, MA, USA), and peripheral oxygen saturation was assessed with MouseOx Plus pulse oximetry (STARR Life Sciences, Oakmont, PA, USA). Following ventilation, anesthesia was reversed with atipamezole (1.5 mg/kg) and mice were extubated.

At 4 h post-extubation, mice were re-anesthetized with ketamine/dexmedetomidine. Blood was drawn from the inferior vena cava and bronchoalveolar lavage (BAL) was performed to collect BAL fluid (BALF). Mice underwent cardiac perfusion with 20 mL of PBS + 0.5 mM EDTA. Brains were harvested and dissected into cortical and hippocampal (inner brain) regions, then flash frozen.

### SomaScan proteomics

2.4 µg of soluble brain tissue lysate or 55 µL plasma per sample were analyzed by SomaScan proteomics using 96-well plates on a Tecan Fluent 780 liquid handling robot at the BIDMC Genomics, Proteomics, Bioinformatics and Systems Biology Center using the 7k SomaScan v4.1 Assay Kit for human plasma (SomaLogic, Inc., Boulder, CO) for the plasma samples that measures expression of 7,307 proteins and the 11k SomaScan v5.0 Assay-Human Cell & Tissue Kit (Product Code 900 − 0057) for the brain tissue samples that measures expression of 10,778 proteins, respectively, using highly selective single-stranded modified Slow Off-rate Modified DNA Aptamers (SOMAmer) according to the manufacturer’s standard protocol. Whole cell lysates from tissue samples obtained from isolated frozen mouse cortical and hippocampal (inner brain) regions were generated in T-PER (Thermo) supplemented with 1x HALT protease inhibitor cocktail using a TissueLyser II (Qiagen). Five pooled plasma Calibrator replicates, 3 pooled plasma Quality Control replicates, and 3 buffer replicates were used to control for batch effects for the plasma samples. Three kit provided pooled cell lysate Calibrator replicates and three no protein background controls were used to normalize and calibrate the brain tissue lysate samples and to estimate accuracy, precision, and buffer background. Twelve hybridization controls and 296 non-human SOMAmers were added alongside the 7,307 or 10,778 SOMAmers, respectively, to control for readout variability for each set of 85 test samples. Sample-to-sample variability was further controlled by several hybridization spike-in controls. Hybridization normalization, plate scaling, median normalization, and calibration of the SomaScan data, performed according to the standard quality control (QC) protocols at SomaLogic, demonstrated that all samples passed the established QC criteria and were fit for further analysis. Although SomaScan panels are optimized for human targets, prior studies demonstrate substantial cross-reactivity with murine orthologs and support SomaScan for discovery-level proteomic profiling in mice. Species-dependent differences in target sequence, epitope accessibility, or aptamer affinity may affect individual measurements. Nevertheless, recent systematic evaluation of the SomaScan 7 K platform by SomaLogic shows that more than 80% of SOMAmer reagents generate measurable signals exceeding technical assay variation and detection of biologically meaningful protein variation in murine plasma samples across multiple mouse strains. Platform quality-control procedures establish technical assay performance but do not independently establish target specificity in murine tissue.

### Statistical and pathway analysis

#### Software and general approach

All statistical analyses were performed using R version 4.2.2. Data were analyzed in raw form without transformation unless otherwise specified.

#### Differential protein expression analysis

Differential protein expression between VILI and SB groups was assessed using two-sample t-tests. For each protein, mean and median values were calculated for both groups after removing missing values. T-test statistics and corresponding p-values were computed after log2 transformation of the raw relative fluorescent unit (RFU) values, followed by Benjamini-Hochberg (BH) correction to control for multiple testing. Due to the small sample size, no significant (BH *p* < 0.05) proteins were found and we proceeded with nominal p-values. Fold changes were calculated as the ratio of group means and medians. Statistical significance was defined as *p* < 0.05.

#### Boxplot analysis

Box whisker plots of median protein expression values were created using XLSTAT (Addinsoft, Long Island City, NY).

#### Volcano plot analysis

Volcano plot analysis was performed to visualize the relationship between fold change magnitude and statistical significance. Median fold changes were calculated between VILI and SB groups. Proteins were classified into three categories: significantly upregulated (log2 fold change > log2(1.2) and *p* < 0.05), significantly downregulated (log2 fold change < -log2(1.2) and *p* < 0.05), or non-significant. The volcano plot displays log2 fold change on the x-axis and -log10 p-value on the y-axis. The top 20 most significant proteins with lowest nominal p-values were labeled.

#### Principal component analysis (PCA)

Three-dimensional Principal Component Analysis was performed using the top 20 most significant proteins identified from the differential expression analysis. Protein selection was based on statistical ranking. Prior to PCA, all protein expression values were standardized using z-score normalization (scale=TRUE) to ensure equal contribution regardless of expression magnitude. PCA was computed using the prcomp() function, and the first three principal components (PC1, PC2, PC3) were extracted for three-dimensional visualization using the plotly package.

#### Hierarchical clustering heatmap

A hierarchical clustered heatmap was generated using the pheatmap package. Clustering was performed using Pearson correlation distance with complete linkage for both proteins and samples to identify patterns of co-expression and sample relationships.

#### Venn diagrams

Venn diagrams were constructed to visualize the overlap of significant proteins between the comparisons at the raw p-value (*p* < 0.05) level using InteractiVenn [12].

#### Ingenuity pathway analysis

To assess for potential biological pathways underlying the differentially expressed mouse brain and plasma protein signatures and to more precisely understand the complex interactions between the differentially expressed proteins, we performed functional category, canonical pathway, interactive network, and upstream regulator analyses of dysregulated proteins in VILI compared to SB, with *p* < 0.01 for the hippocampal brain tissue and with *p* < 0.05 and absolute fold change (FC) > 1.3 for plasma, using the Ingenuity Pathway Analysis (IPA) software tool (QIAGEN, Redwood City, CA), a repository of biologic interactions and functions created from millions of individually modeled relationships that range from the molecular (proteins, genes) to organism (diseases) level. IPA upstream-regulator analysis infers candidate regulatory involvement from relationships between observed downstream protein changes and curated interaction networks. These predictions do not directly measure regulator expression or activity and do not establish causal regulatory effects.

## Results

**Fig. 1:**
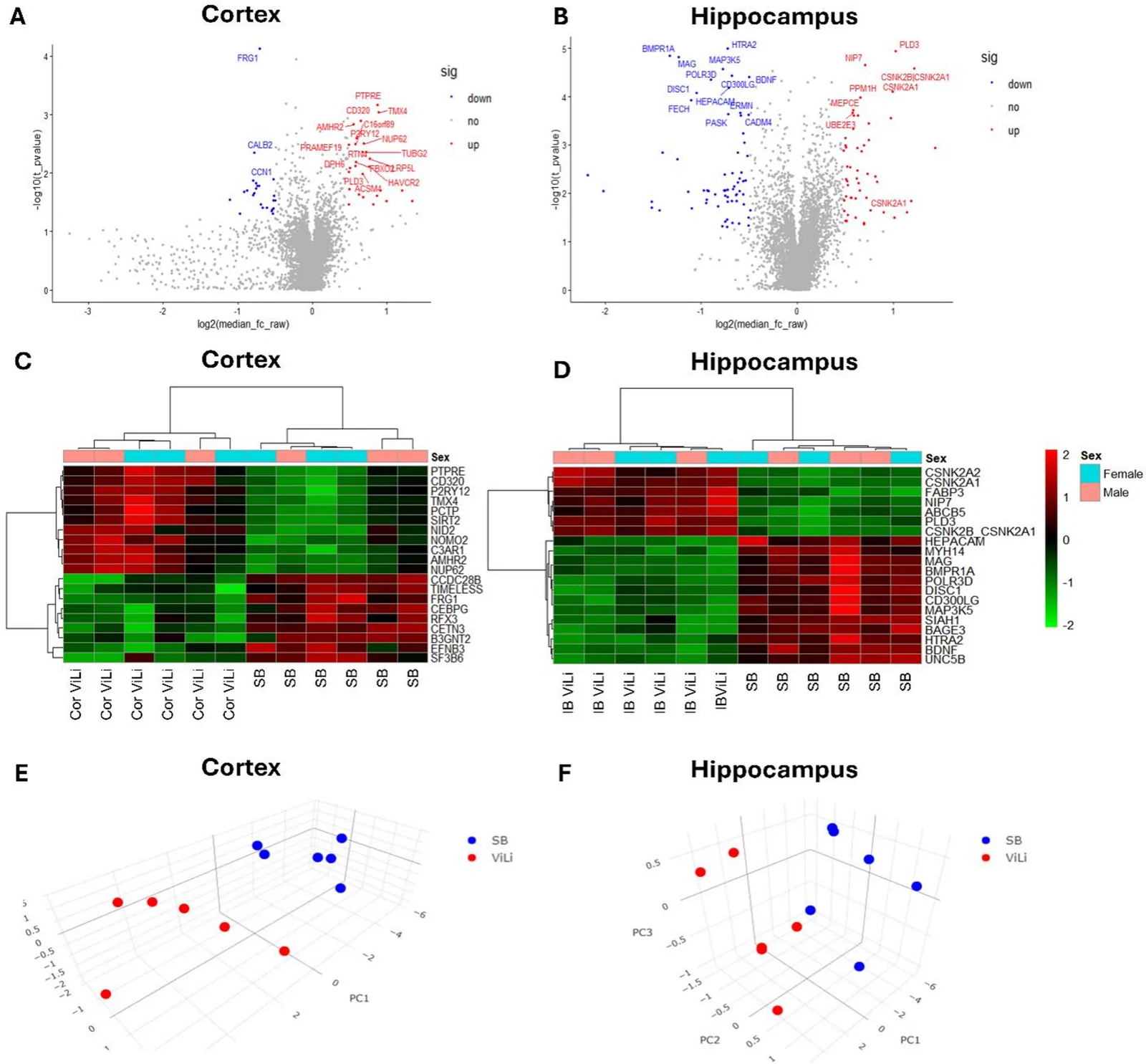
Brain proteomic profiling of VILI and SB mice. Volcano plots of differentially expressed proteins in cortex (**A**) and hippocampus (**B**), with upregulated (red) and downregulated (blue) proteins meeting significance (*p* < 0.05) and fold change (|log2 FC| > log2(1.2)) thresholds. Top 20 proteins labeled. Hierarchical clustering heatmaps of the top 20 differentially expressed proteins in cortex (**C**) and hippocampus (**D**), with sex annotation. Three-dimensional PCA using the top 20 proteins in cortex (**E**) and hippocampus (**F**). *n* = 6 per group. The hippocampus yielded nearly four-fold more significantly altered proteins than the cortex (995 vs. 253), and unsupervised methods achieved complete separation of VILI and SB animals in both regions, confirming robust, condition-driven proteomic signatures. These results suggest that VILI induces region-specific protein changes, disproportionately affecting the hippocampus

**Fig. 2:**
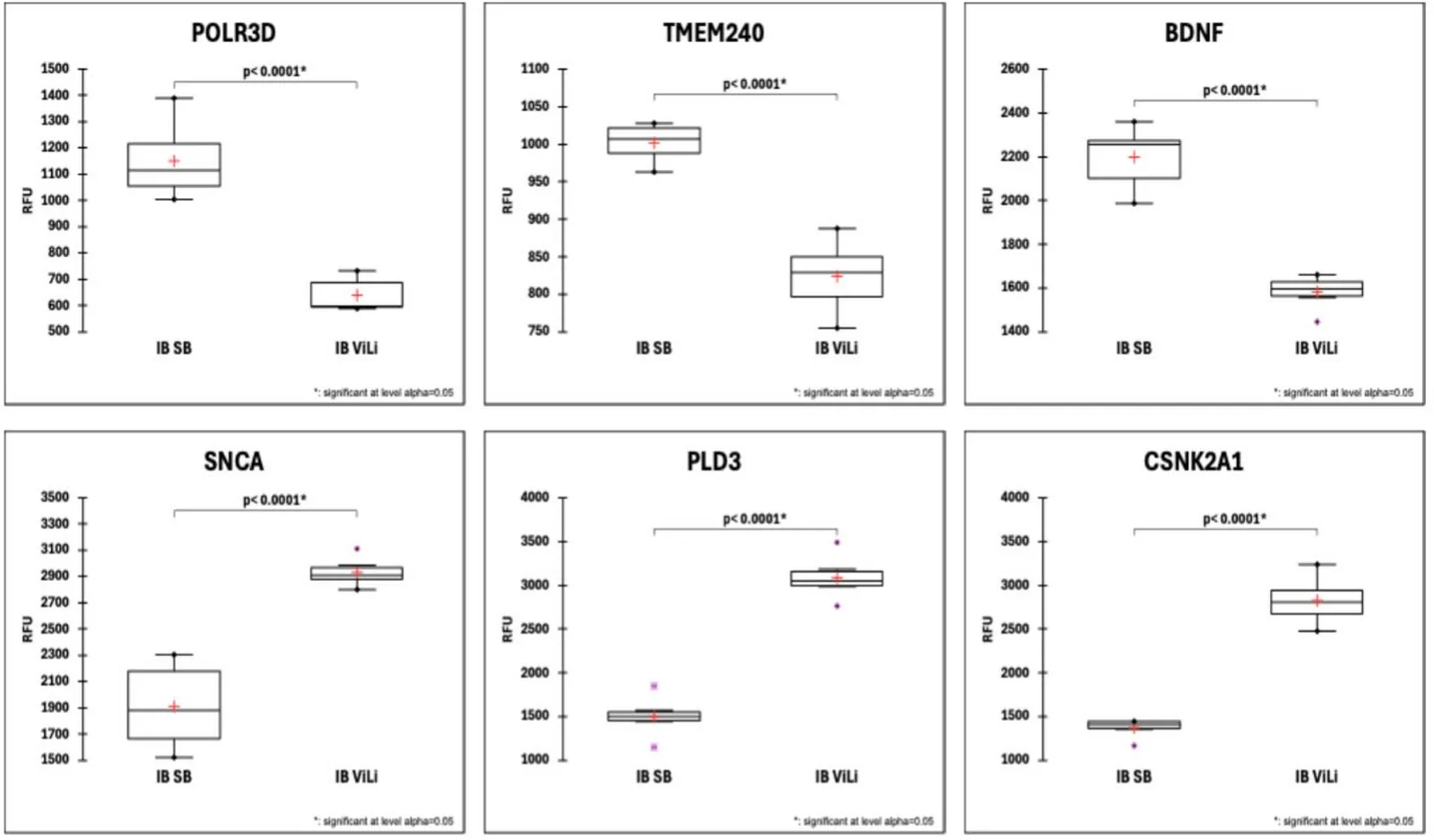
Box whisker plots of selected hippocampal proteins with altered expression in SB vs. VILI. Expression (RFU) of POLR3D, TMEM240, BDNF, SNCA, PLD3, and CSNK2A1 in SB versus VILI hippocampal (IB) specimens. Boxes represent interquartile range; horizontal line indicates median; + indicates mean. *p < 0.05, two-sample t-test. *n* = 6 per group. These proteins span synaptic plasticity (BDNF, SNCA), neuroinflammatory signaling (CSNK2A1), and Alzheimer’s disease risk (PLD3), collectively implicating early neurodegenerative molecular programs. These results suggest that VILI may induce hippocampal protein changes implicated in neurodegenerative processes

SomaScan proteomics identified 253 and 995 proteins with significantly different expression levels (*p* < 0.05) between VILI and SB mice in the cortex and hippocampal regions, respectively. Volcano plots illustrate the relationship between statistical significance (-log10-transformed p-values) and effect size (log2 fold changes) (Fig. 1A and B). The 20 proteins with the most significant expression differences between VILI and SB mice in each brain region are shown in Fig. 1C-D and showed separation between VILI and SB by hierarchical clustering, an unsupervised learning approach, within this discovery cohort. Principal component analysis in 3 dimensions using the top 20 proteins in each region likewise showed separation between the SB and VILI animals for hippocampal and cortical brain regions (Fig. 1E-F). Comparisons of selected proteins from the analysis of the hippocampal specimens are shown in box whisker plots in Fig. 2. Among the most significantly altered proteins in hippocampal VILI specimens were CSNK2A1, SNCA, POLR3D, PLD3, BDNF, and TMEM240. It is worth noting that no individual proteins remained significant after Benjamini-Hochberg correction for multiple testing.

**Fig. 3:**
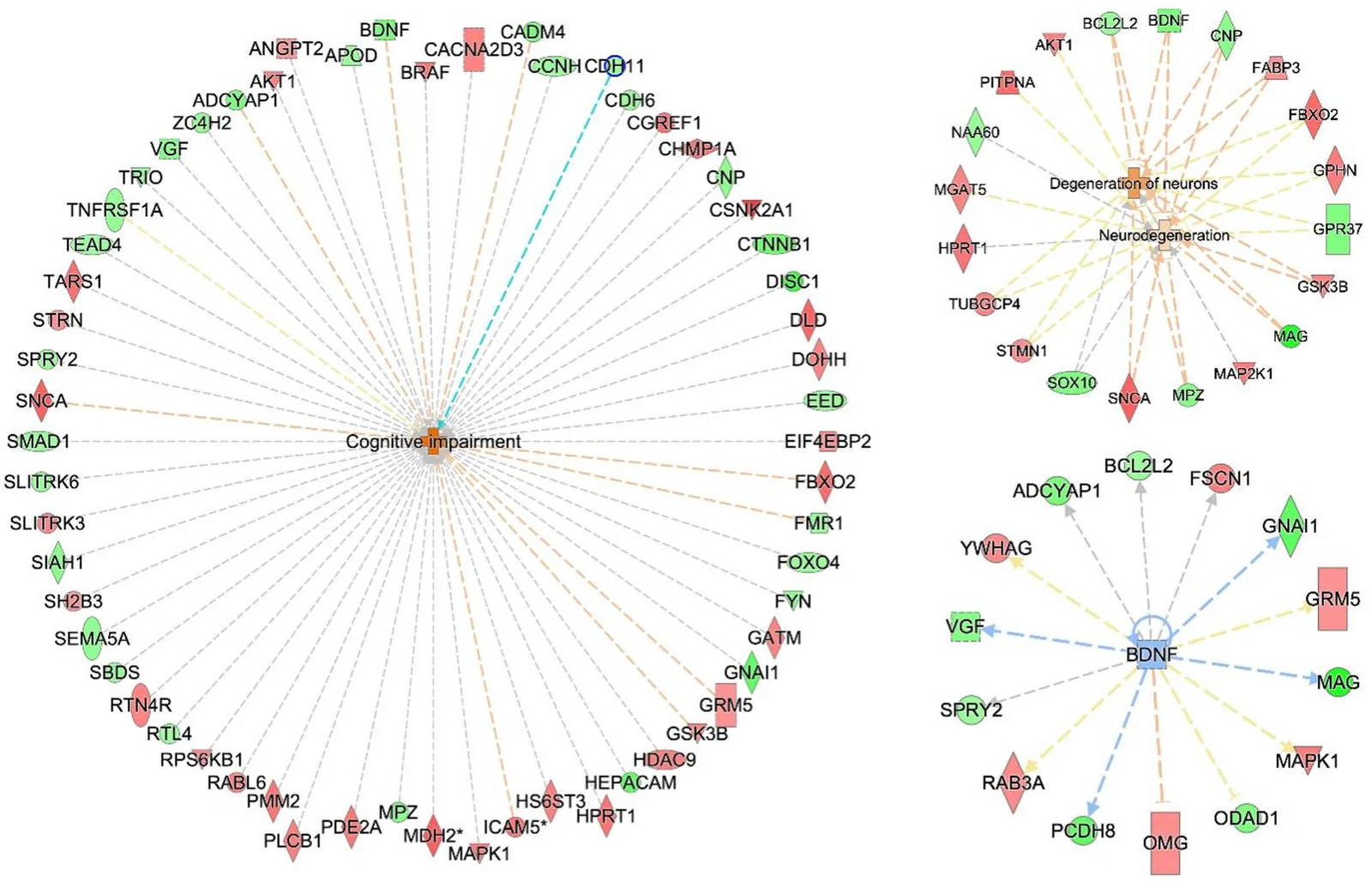
Ingenuity Pathway Analysis of hippocampal VILI specimens (*p* < 0.01). Biofunctional enrichment network for cognitive impairment (left). Biofunctional network for degeneration of neurons and neurodegeneration (top right). BDNF interaction network (bottom right). Node color reflects measured expression change (red/pink = upregulated, green = downregulated). See Supplemental Fig. 1 for additional hippocampal networks. Co-enrichment of degenerative and maturation-related categories suggests that VILI may induce a dual injury mechanism: direct neuronal damage alongside impaired regenerative capacity

Across hippocampal VILI specimens, pathway and network analyses identified two dominant biological themes (Fig. 3): (1) injury–inflammation programs mapping onto annotations related to neurodegeneration and cognitive impairment, and (2) concurrent shifts in differentiation and maturation programs. Biofunctional enrichment networks highlighted proteins associated with neuronal stress, synaptic dysfunction, and glial biology, including BDNF, CTNNB1, SOX9, SOX10, SNCA, GSK3B, MAPK1 [13–16]. Functional categories enriched in this analysis include “degeneration of neurons,” “neurodegeneration,” and “cognitive impairment.” Enrichment of differentiation and maturation-related categories, including differentiation of neural precursor cells and maturation of neurons, was also observed.

Upstream regulator analysis identified candidate master regulators whose downstream targets significantly overlapped with the observed proteomic shifts. Predicted regulators included CTNNB1 (β-catenin/Wnt signaling), BMP7, GABA, BDNF, MYCN, PTEN, SOX9, NR3C1 (glucocorticoid receptor), SNCA, and PPARGC1A (PGC-1α).

**Fig. 4:**
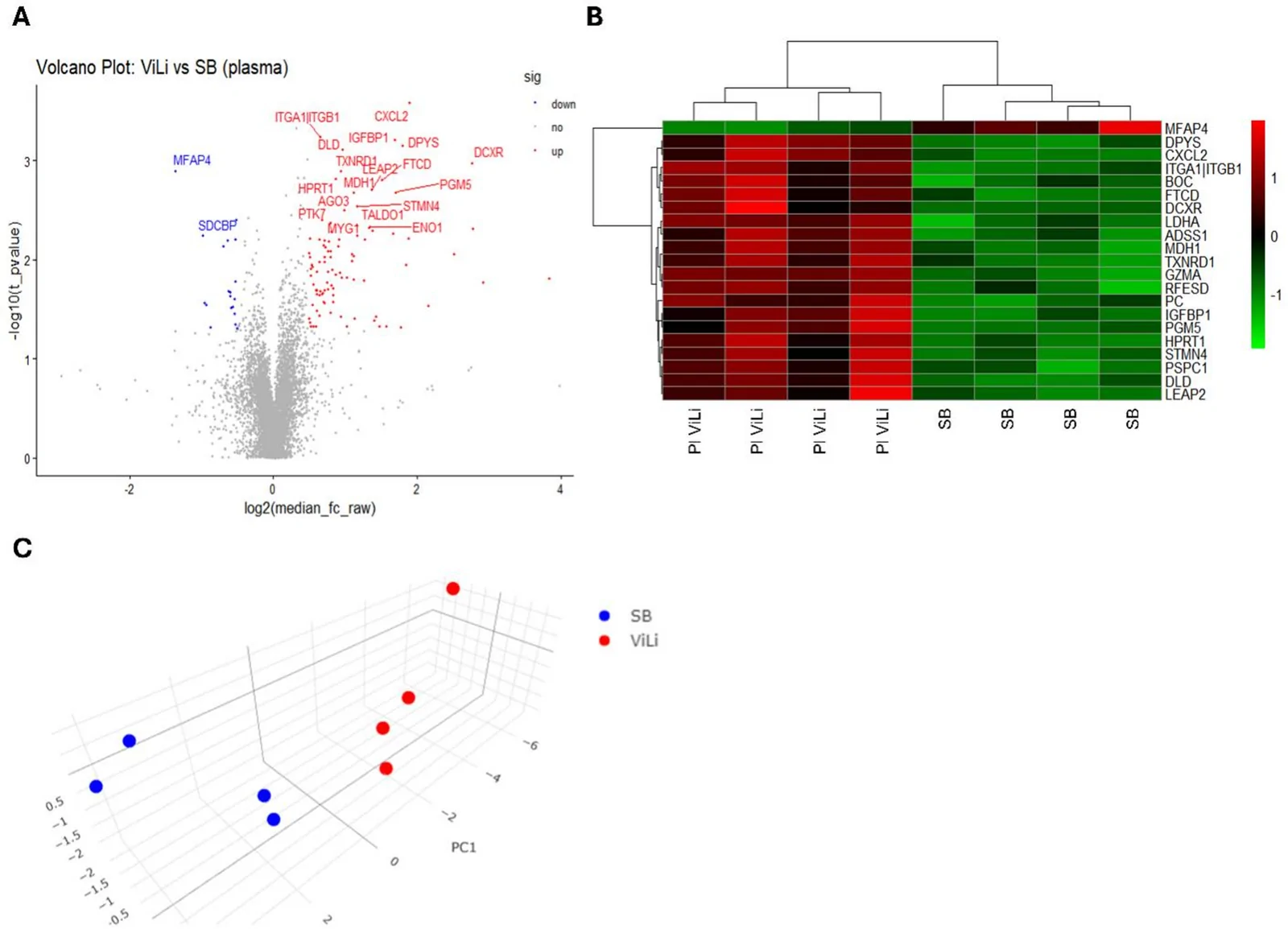
Plasma proteomic profiling of VILI and SB mice. (**A**) Volcano plot of differentially expressed plasma proteins. (**B**) Hierarchical clustering heatmap of the top 20 differentially expressed plasma proteins. (**C**) Three-dimensional PCA using the top 20 plasma proteins. Notation as in Fig. 1. *n* = 4 per group. The plasma proteomic response encompasses inflammatory, metabolic, and vascular injury mediators, indicating that VILI generates a coordinated peripheral injury signature that may serve as the upstream signal driving the observed brain proteomic changes

**Fig. 5:**
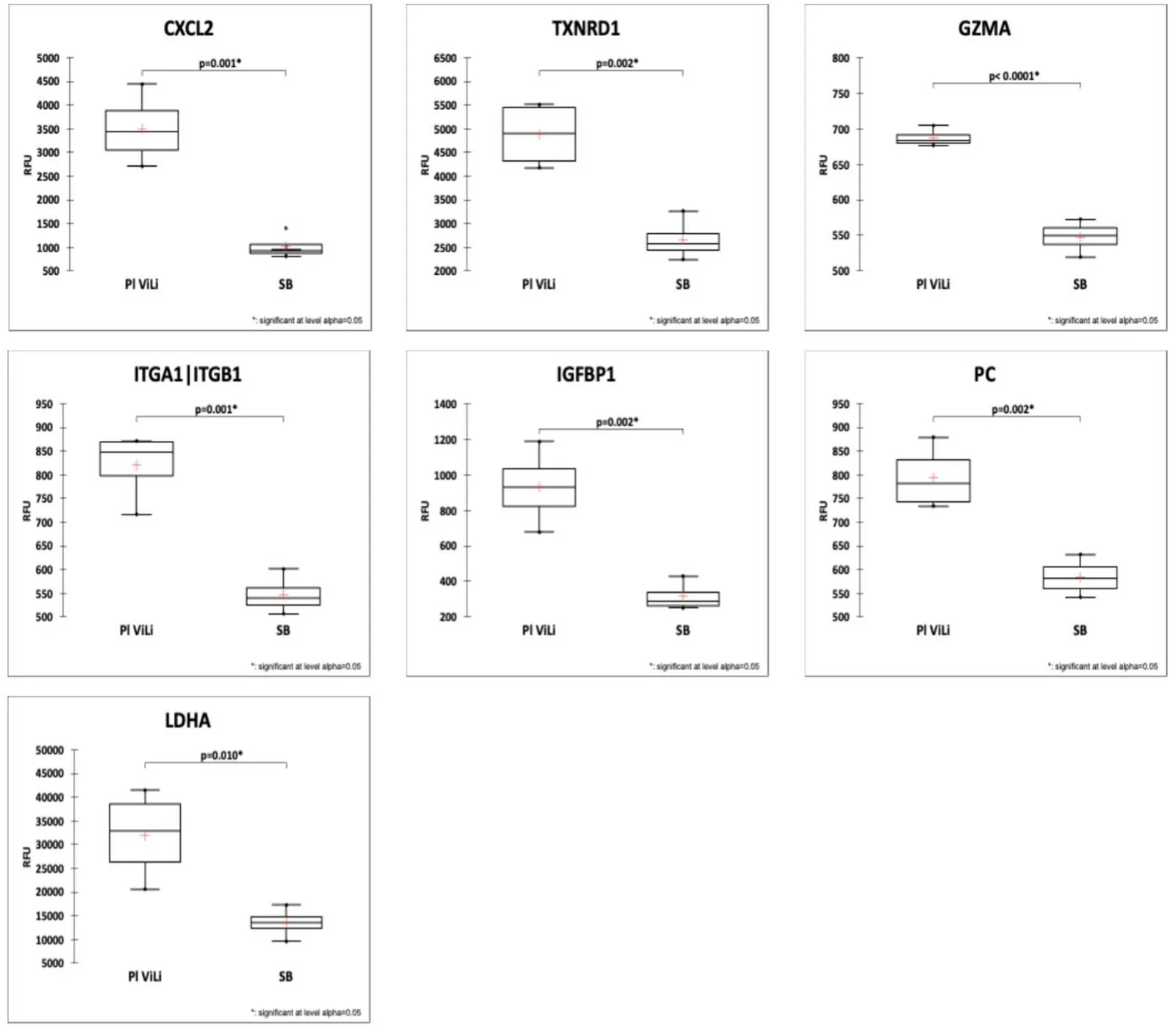
Box whisker plots of selected plasma proteins with altered expression in VILI vs. SB. Expression (RFU) of CXCL2, TXNRD1, GZMA, ITGA1|ITGB1, IGFBP1, PC, and LDHA in VILI versus SB plasma. Notation as in Fig. 2. *n* = 4 per group. These results suggest that VILI induces alterations in downstream protein targets of IL-6 signaling (CXCL2, GZMA, IGFBP1), while LDHA and PC reflect a glycolytic–gluconeogenic shift characteristic of acute systemic inflammation

To identify differentially expressed proteins that discriminate between VILI and SB in plasma, t-test results used to analyze SomaScan data for 7,307 proteins were visualized by a Volcano plot (Fig. 4A). SomaScan identified 290 plasma proteins with significantly different (*p* < 0.05) expression levels between VILI and SB mice (Fig. 4A). Hierarchical clustering (Fig. 4B) of the 20 proteins with the most significant differences showed clear segregation of samples by condition. Principal component analysis using these 20 plasma proteins reveals a similar separation between VILI and SB groups in 3 dimensions (Fig. 4C). Comparisons using box whisker plots of selected plasma proteins implicated in inflammatory, metabolic, and vascular injury processes are shown in Fig. 5. Among the most significantly altered were CXCL2, GZMA, TXNRD1, IGFBP1, PC, LDHA, and ITGA1/ITGB1.

**Fig. 6:**
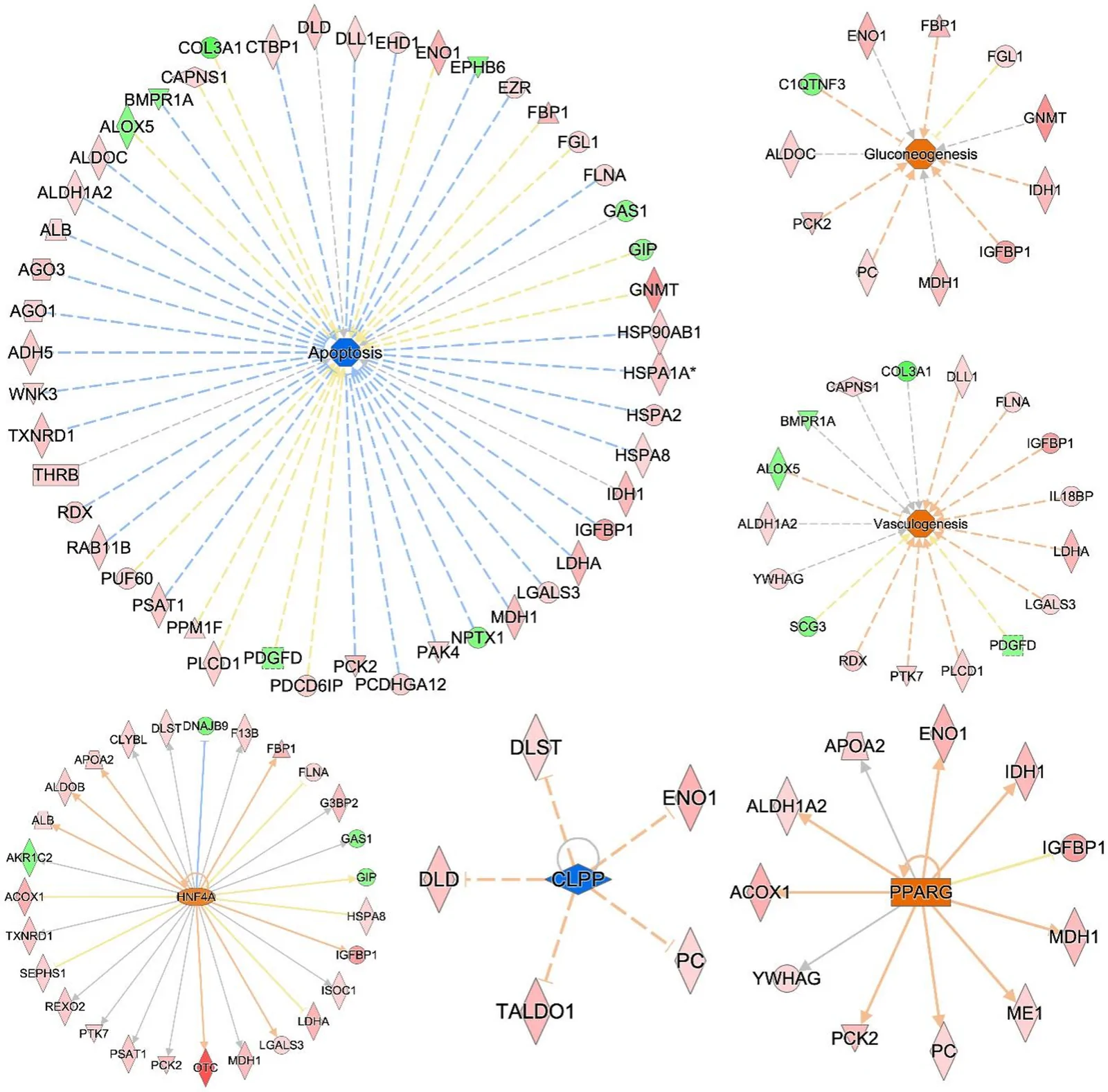
Ingenuity Pathway Analysis of VILI plasma specimens (*p* < 0.05, |FC| > 1.3). Biofunctional networks for apoptosis (top left), gluconeogenesis (top right), and vasculogenesis (middle right). Upstream regulator networks for HNF4A (bottom left), CLPP (bottom center), and PPARG (bottom right). Node color reflects measured expression change; regulator color indicates predicted activation state (orange = activated, blue = inhibited). See Supplemental Fig. 2 for PKM upstream regulator network. These results suggest that VILI may induce systemic metabolic reprogramming consistent with IL-6-mediated acute phase physiology, as evidenced by predicted activation of HNF4A and PPARG alongside inhibition of CLPP

Pathway and network analyses of the VILI plasma specimens (Fig. 6) identified enrichment of functional categories including gluconeogenesis, vasculogenesis, and apoptosis. Biofunctional networks centered on FBP1, PCK2, PC, LDHA, CXCL2, ALDOB, and IGFBP1. Upstream regulator analysis predicted enhanced activity of HNF4A, PPARG, and PKM, along with decreased activity of the mitochondrial endopeptidase CLPP.

**Fig. 7:**
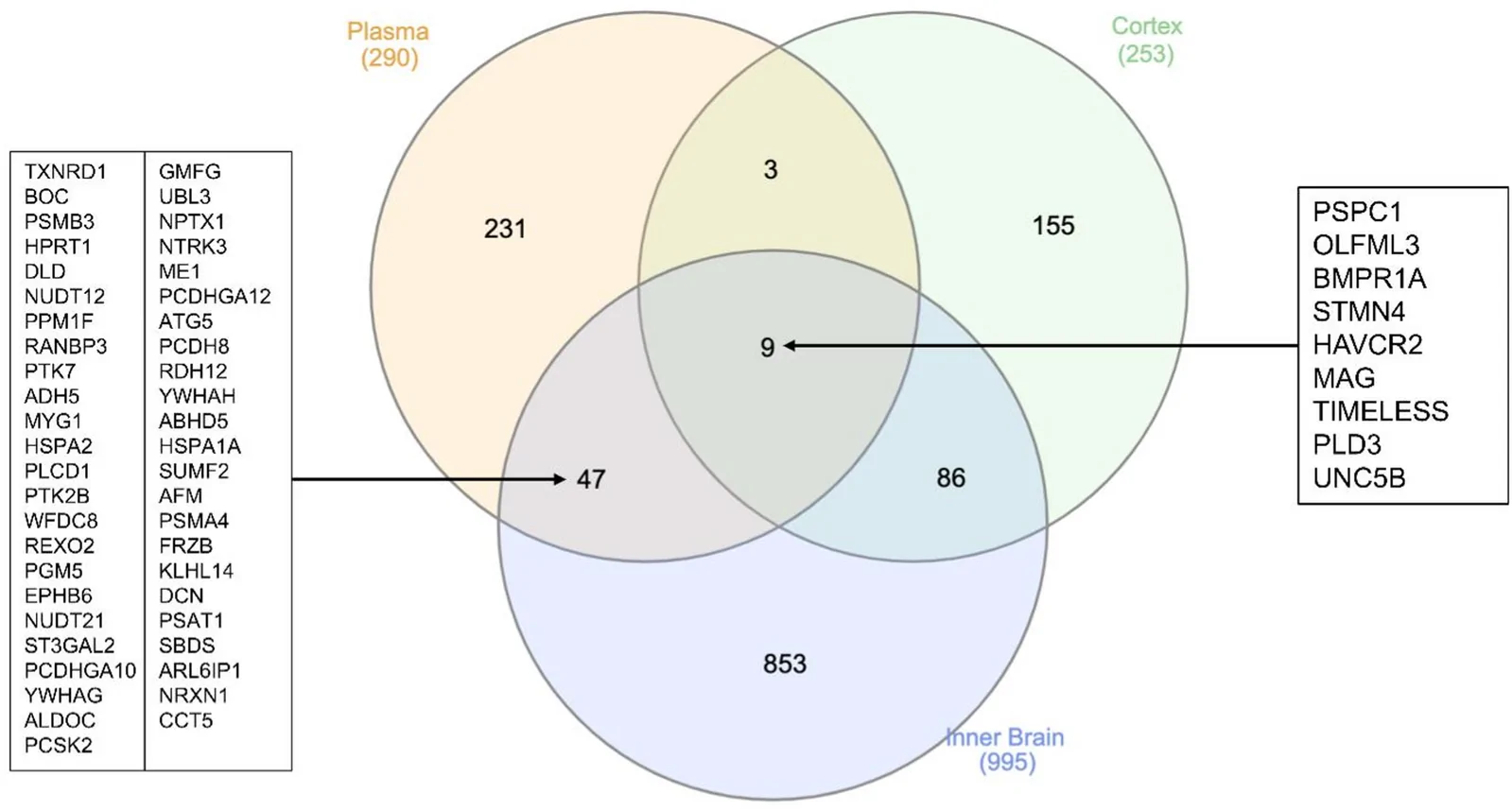
Venn diagram of significantly altered proteins (*p* < 0.05) across plasma (290), cortex (253), and hippocampal (995) compartments. Nine proteins shared across all three compartments are listed (right). Forty-seven proteins overlapping between plasma and hippocampus are listed (left). The hippocampus demonstrated the most extensive proteomic disruption of any compartment, and the 9 cross-compartment proteins include brain-enriched targets (MAG, STMN4) and the Alzheimer’s risk gene PLD3. Their detection in plasma suggests that VILI may induce blood–brain barrier compromise and provides a molecular link between peripheral and central injury

Venn diagram analysis (Fig. 7, *p* < 0.05) showed that the hippocampal region exhibited the greatest number of significantly altered proteins (*n* = 995), exceeding cortex (*n* = 253) and plasma (*n* = 290). Only 9 proteins (PSPC1, OLFML3, BMPR1A, STMN4, HAVCR2, MAG, TIMELESS, PLD3, UNC5B) overlapped across all three compartments, indicating predominantly region- and compartment-specific molecular responses. 47 proteins overlapped between plasma and hippocampus.

## Discussion

The proteomic signatures identified in this study reveal that VILI initiates a coordinated disruption of molecular pathways associated with and believed to be fundamental to neuronal survival, synaptic integrity, and cognitive function. The hippocampus -- central to learning and memory -- showed the most extensive proteomic remodeling, suggesting that systemic injury signals generated during VILI may preferentially converge on neural circuits governing cognition. Mechanistically, the altered proteins and pathways map onto canonical neurodegenerative processes, suggesting that VILI may trigger early molecular events analogous to those observed in Alzheimer’s disease, Parkinson’s disease, and stress-related cognitive decline. While these shared pathways do not establish that VILI directly induces neurodegenerative disease, they point to a common molecular vulnerability centered on synaptic integrity and stress-related neurological dysfunction that may contribute to subsequent cognitive decline. These findings are further consistent with clinical studies that demonstrate increased risk of Alzheimer’s disease and related dementias in patients with acute respiratory distress syndrome, a severe acute lung injury phenotype [17–20].

A dominant mechanistic theme emerging from the hippocampal data is synaptic dysfunction driven by perturbed neurotrophic and Wnt/β-catenin signaling. Reduced or dysregulated expression of BDNF, a master regulator of synaptic plasticity, long-term potentiation, and memory encoding, suggests impaired trophic support for hippocampal neurons [14]. Concurrent alterations in CTNNB1 and GSK3B imply destabilization of Wnt/β-catenin pathways, which are essential for dendritic maintenance, adult neurogenesis, and protection against excitotoxic injury [13, 21]. Disruption of these pathways is a well-established driver of cognitive impairment, positioning them as potential mechanistic nodes in VILI-associated brain dysfunction.

The identification of SNCA dysregulation further demonstrates overlap with neurodegenerative pathways. α-Synuclein accumulation is a hallmark of synaptic toxicity and impaired vesicle cycling, and its altered expression in VILI suggests that mechanical lung injury may propagate systemic signals capable of perturbing presynaptic homeostasis. Additional changes in proteins such as MAPK1, SOX9, and SOX10 point toward activation of stress-responsive kinase cascades and glial transcriptional programs that promote neuroinflammation, astrogliosis, and impaired neuronal maturation. These processes collectively weaken synaptic networks and reduce the brain’s capacity for repair.

Pathway enrichment analyses support this potential mechanistic interpretation. Categories such as “degeneration of neurons,” “neurodegeneration,” and “cognitive impairment” reflect activation of conserved injury programs that integrate mitochondrial dysfunction, oxidative stress, and impaired proteostasis. The parallel enrichment of pathways related to neural precursor differentiation and neuronal maturation suggests that VILI not only damages existing neurons but also disrupts the regenerative capacity of hippocampal circuits. This dual hit -- synaptic injury plus impaired neurogenesis -- is a plausible mechanism underlying persistent cognitive deficits after critical illness.

Upstream regulator analyses provide additional insights. Predicted involvement of NR3C1 (glucocorticoid receptor) suggests that stress-hormone signaling may amplify neuronal vulnerability, consistent with the cognitive consequences of systemic inflammation and critical illness while predicted involvement of regulators such as PTEN, PPARGC1A (PGC-1α), and BMP7 is consistent with pathways related to mitochondrial stress, impaired metabolic resilience, and altered neurodevelopmental signaling – pathways that are increasingly recognized as early drivers of neurodegenerative cascades.

Within the hippocampal region, the distinct proteomic signature suggests the active propagation of IL-6 signaling and its downstream consequences on synaptic plasticity. The significant dysregulation of Casein Kinase 2 (CSNK2A1, CSNK2B) is particularly notable, given its role in constitutively phosphorylating STAT3 and modulating NF-κB activity [22]. An upregulation or dysregulation of these kinase networks may serve to amplify or prolong the IL-6/STAT3 signaling cascade within local glial and neuronal populations. This sustained neuroinflammatory signaling provides a plausible molecular basis for the observed suppression of BDNF and the dysregulation of synaptic regulators like TMEM240. Because elevated central IL-6 and prolonged STAT3 activation are established negative regulators of neurotrophic expression, this cascade offers a compelling link between peripheral lung injury and the acute synaptic uncoupling characteristic of delirium and neurodegeneration. This is further supported by recent evidence that peripheral IL-6 administration is sufficient to exacerbate hippocampal amyloid-β deposition and microglial activation in the context of pre-existing neurodegenerative pathology, establishing a direct mechanistic link between circulating IL-6 and central neurodegenerative processes [23]. More broadly, upstream regulator analysis identified potential predicted upstream master regulators (CTNNB1, NR3C1, SNCA, BDNF, SOX9, and PPARGC1A) that converge on synaptic plasticity, neuronal stability, glial differentiation, and mitochondrial/metabolic regulation [14, 21, 24]. The concurrent predicted involvement of stress-response (NR3C1) and energy metabolism (PPARGC1A) regulators alongside neuroinflammatory mediators is consistent with a model of IL-6-linked signaling interacting with stress-axis and metabolic pathways to contribute to hippocampal dysfunction [25].

When integrated with prior human neuropathologic studies, the mouse hippocampal VILI-induced delirium proteomic profile aligned with established neuroinflammatory and glial activation signatures. Postmortem analyses of delirium cases demonstrated increased microglial activation (HLA-DR, CD68), elevated astrocytic reactivity (glial fibrillary acidic protein), and increased IL-6 immunoreactivity across brain regions, supporting an IL-6–weighted inflammatory mechanism [26]. Population-based clinicopathologic studies further showed that delirium interacts with dementia pathology burden to accelerate cognitive decline, suggesting that acute inflammatory perturbations may amplify underlying neurodegenerative processes [27]. Glial-centered models of delirium emphasize astrocytic metabolic dysfunction, microglial activation, BBB disruption, and maladaptive synaptic remodeling, mechanisms that are consistent with the proteomic programs observed in the present dataset [28]. The hippocampal regions demonstrated the most pronounced proteomic alterations, underscoring region-specific vulnerability. This pattern parallels Alzheimer’s disease, where hippocampal circuits are preferentially affected.

Plasma proteomics further supports a systemic-to-central mechanism. Altered circulating levels of CXCL2, GZMA, TXNRD1, IGFBP1, LDHA, and other inflammatory and metabolic mediators suggest that VILI generates a peripheral injury signature capable of crossing or modulating the blood–brain barrier [29–33]. Although only nine proteins overlapped across plasma, cortex, and hippocampus, several of these—such as PLD3, BMPR1A, and UNC5B—are themselves implicated in neurodegenerative biology, raising the possibility that circulating factors may act as upstream triggers for brain-specific injury programs [34–36].

While our findings provide novel mechanistic insights into the lung-brain axis, several limitations must be acknowledged. First, our proteomic analysis captures a single, acute timepoint following mechanical ventilation, which aligns with the onset of acute delirium-like phenotypes but does not track the progression from acute injury to chronic neurodegeneration [37]. Furthermore, we use a model of isolated acute lung injury induced by high tidal volume mechanical ventilation, which does not necessarily reflect current clinical practice favoring lower tidal volume strategies. However, in prior studies, we have demonstrated that this model induces structural and functional phenotypes of acute and chronic neurodegenerative processes through systemic inflammatory signaling [7–9]. Such signaling is often amplified in the context of common clinical precipitants of mechanical ventilation, including underlying inflammatory states or restrictive pulmonary environments, even when lower tidal volume strategies are used. The overlap between acutely dysregulated proteins and those implicated in chronic neurodegenerative pathways (e.g., SNCA, BDNF, GSK3B) is notable and suggests that some acute-phase molecular events may seed or accelerate longer-term processes, requiring testing with longitudinal studies. Second, utilizing bulk tissue homogenates precludes the assignment of these proteomic shifts to specific cellular subtypes. While pathway analyses strongly implicate microglial and astrocytic networks, single-cell resolution is required to fully map the spatial dynamics of the glial response. Third, the SomaScan Assay is optimized for human targets, and its application to murine tissue relies on evolutionary conservation of the targeted epitopes. The possibility of differential aptamer affinity across species should be considered when interpreting effect sizes. Further, the modest sample sizes (*n* = 4 per group for plasma, *n* = 6 per group for brain) are typical for discovery-phase proteomics but limit statistical power. We recognize that these small sample sizes inherently increase susceptibility to both false-positive results driven by individual sample variability and false-negative rates where true proteomic shifts fail to reach significance. Finally, we acknowledge that no individual proteins met significance after correction for multiple hypothesis testing and nominal p-values were used. As such, this study’s results should be viewed as exploratory and hypothesis generating and require validation in independent cohorts and by orthogonal methods. Despite these limitations, the robust compartmentalization and strong alignment with known human neuroinflammatory signatures underscore the translational relevance of this model and emphasize the need for tissue-based approaches to define disease mechanisms.

Taken together, these findings support a model in which VILI induces a multi-compartment injury response that extends beyond the lung to engage molecular programs associated with cognitive impairment and neurodegeneration. These data highlight the need for mechanistic studies to define how systemic injury signals propagate to the brain and call for future studies to evaluate whether targeted disruption of these pathways rescues neurodegenerative changes. Future studies are needed to evaluate the long-term evolution of the observed proteomic signatures and their relationship to long-term cognitive function. Finally, future studies are also indicated to evaluate peripheral markers of acute neurodegeneration, which may provide diagnostic and prognostic value in clinical settings.

## Conclusions

VILI induces a multi-compartment proteomic injury response characterized by a dominant hippocampal signature enriched for neurodegeneration, cognitive impairment, and disrupted neuronal maturation, with parallel plasma shifts consistent with IL-6-associated inflammatory-metabolic stress and blood-brain barrier compromise. These findings support a model in which peripheral lung injury activates coordinated neuroinflammatory pathways converging on early neurodegenerative mechanisms, highlighting the lung-brain axis as a critical therapeutic target in patients undergoing mechanical ventilation.

## Supplementary Material

Supplementary Material 1.

## Data Availability

The datasets supporting the conclusions of this article are available from the corresponding author upon reasonable request.

## Abbreviations

ACOX1: Acyl-CoA oxidase 1
ADSS1: Adenylosuccinate synthetase isozyme 1
AKT1: AKT serine/threonine kinase 1
ALDOB: Aldolase B
BAL: Bronchoalveolar lavage
BALF: Bronchoalveolar lavage fluid
BBB: Blood-brain barrier
BDNF: Brain-derived neurotrophic factor
BH: Benjamini-Hochberg
BMP7: Bone morphogenetic protein 7
BMPR1A: Bone morphogenetic protein receptor type 1 A
CLPP: Caseinolytic mitochondrial matrix peptidase proteolytic subunit
CSNK2A1 (CK2α): Casein kinase 2 alpha 1
CSNK2B (CK2β): Casein kinase 2 beta
CTNNB1: Catenin beta 1 (β-catenin)
CXCL2: C-X-C motif chemokine ligand 2
DCXR: Dicarbonyl and L-xylulose reductase
ENO1: Enolase 1
FBP1: Fructose-bisphosphatase 1
FMR1: Fragile X messenger ribonucleoprotein 1
GNAI1: G protein subunit alpha i1
GRM5: Glutamate metabotropic receptor 5
GSK3B: Glycogen synthase kinase 3 beta
GZMA: Granzyme A
HAVCR2 (TIM-3): Hepatitis A virus cellular receptor 2 (T-cell immunoglobulin and mucin domain-containing protein 3)
HNF4A: Hepatocyte nuclear factor 4 alpha
IGFBP1: Insulin-like growth factor binding protein 1
IL-6: Interleukin-6
IPA: Ingenuity Pathway Analysis
ITGA1: Integrin subunit alpha 1
ITGB1: Integrin subunit beta 1
LDHA: Lactate dehydrogenase A
MAG: Myelin-associated glycoprotein
MAPK1: Mitogen-activated protein kinase 1
MDH1: Malate dehydrogenase 1
MFAP4: Microfibril-associated protein 4
MYCN: N-myc proto-oncogene protein
NR3C1: Nuclear receptor subfamily 3 group C member 1 (glucocorticoid receptor)
OLFML3: Olfactomedin-like 3
PC: Pyruvate carboxylase
PCK2: Phosphoenolpyruvate carboxykinase 2
PCA: Principal component analysis
PEEP: Positive end-expiratory pressure
PKM: Pyruvate kinase M
PLD3: Phospholipase D3
POLR3D: RNA polymerase III subunit D
PPARG: Peroxisome proliferator-activated receptor gamma
PPARGC1A (PGC-1α): Peroxisome proliferator-activated receptor gamma coactivator 1 alpha
PSPC1: Paraspeckle component 1
PTEN: Phosphatase and tensin homolog
RFU: Relative fluorescence units
SB: Spontaneously breathing
SNCA: Alpha-synuclein
SOX9: SRY-box transcription factor 9
SOX10: SRY-box transcription factor 10
STAT3: Signal transducer and activator of transcription 3
STMN4: Stathmin 4
TALDO1: Transaldolase 1
TCF7L2: Transcription factor 7-like 2
TIMELESS: Timeless circadian regulator
TMEM240: Transmembrane protein 240
TXNRD1: Thioredoxin reductase 1
UNC5B: Unc-5 netrin receptor B
VILI: Ventilator-induced lung injury
