# Supplementary material for: Experimental model of ventilator-induced lung injury induces proteomic signatures of neurodegeneration in the hippocampus
Source: Intensive Care Med Exp. 2026 Sep 29;14:128. doi: 10.1186/s40635-026-00981-7 (PMC13624254; doi:10.1186/s40635-026-00981-7)
Supplement: Supplementary file 1 [file 40635_2026_981_MOESM1_ESM.docx]

**Supplemental Figure 1.** Additional hippocampal IPA networks (p < 0.01). Upstream regulators SOX9 and TCF7L2 with downstream functional annotations including differentiation of neural stem/precursor cells, maturation of nervous tissue, and activation of DNA endogenous promoter (top). Differentiation and maturation network (bottom left). SNCA interaction network (bottom right). Node color conventions as in Figure 3. These networks suggest that VILI disrupts both existing neuronal circuits along with regenerative and differentiative capacities of the hippocampus.

**Supplemental Figure 2.** PKM upstream regulator network from plasma IPA (p < 0.05, |FC| > 1.3). PKM (predicted activated) with downstream targets including LDHA, FBP1, ENO1, PC, PCK2, TALDO1, and ACOX1. Node color conventions as in Figure 6. The convergence of these targets on glycolytic and gluconeogenic enzymes suggests that VILI induces systemic metabolic reprogramming.
